# Whole genome sequence analyses of thermotolerant *Bacillus* sp. isolates from food

**DOI:** 10.5808/gi.23030

**Published:** 2023-09-27

**Authors:** Phornphan Sornchuer, Kritsakorn Saninjuk, Pholawat Tingpej

**Affiliations:** 1 Department of Preclinical Science, Faculty of Medicine, Thammasat University, Klongluang, Pathum Thani 12120, Thailand; 2 Thammasat University Research Unit in Nutraceuticals and Food Safety, Faculty of Medicine, Thammasat University, Klongluang, Pathum Thani 12120, Thailand; 3 Porcinotec Co., Ltd., Talat Khwan, Mueang Nonthaburi, Nonthaburi 11000, Thailand

**Keywords:** *Bacillus cereus*, foodborne pathogen, thermotolerance, whole genome sequencing

## Abstract

The *Bacillus cereus* group, also known as *B. cereus sensu lato* (*B. cereus s.l.*), is composed of various *Bacillus* species, some of which can cause diarrheal or emetic food poisoning. Several emerging highly heat-resistant *Bacillus* species have been identified, these include *B. thermoamylovorans*, *B. sporothermodurans*, and *B. cytotoxicus* NVH 391-98. Herein, we performed whole genome analysis of two thermotolerant *Bacillus* sp. isolates, *Bacillus* sp. B48 and *Bacillus* sp. B140, from an omelet with acacia leaves and fried rice, respectively. Phylogenomic analysis suggested that *Bacillus* sp. B48 and *Bacillus* sp. B140 are closely related to *B. cereus* and *B. thuringiensis*, respectively. Whole genome alignment of *Bacillus* sp. B48, *Bacillus* sp. B140, mesophilic strain *B. cereus* ATCC14579, and thermophilic strain *B. cytotoxicus* NVH 391-98 using the Mauve program revealed the presence of numerous homologous regions including genes responsible for heat shock in the *dnaK* gene cluster. However, the presence of a DUF4253 domain-containing protein was observed only in the genome of *B. cereus* ATCC14579 while the intracellular protease PfpI family was present only in the chromosome of *B. cytotoxicus* NVH 391-98. In addition, prophage Clp protease-like proteins were found in the genomes of both *Bacillus* sp. B48 and *Bacillus* sp. B140 but not in the genome of *B. cereus* ATCC14579. The genomic profiles of *Bacillus* sp. isolates were identified by using whole genome analysis especially those relating to heat-responsive gene clusters. The findings presented in this study lay the foundations for subsequent studies to reveal further insights into the molecular mechanisms of *Bacillus* species in terms of heat resistance mechanisms.

## Introduction

*Bacillus cereus* is a spore-forming gram-positive rod that is a common contaminant of food and dairy products. *B. cereus* is one of the major foodborne pathogens that is responsible for causing diarrheal or emetic food poisoning. *B. cereus* strains can be divided into distinct groups based on their growth and survival characteristics (i.e., psychrophilic, mesophilic, and thermophilic). Mesophilic strains grow well at 37ºC and can survive at temperatures below 10ºC [1]. In contrast, psychrophilic strains grow effectively at temperatures below 10ºC, but grow poorly at 37ºC [1]. Psychrophilic strains of *B. cereus* are commonly present in chilled or fresh foods [2-4]. However, some *B. cereus* strains can grow between 20–50ºC and this is representative of a cluster of thermophilic strains [5]. *B. cereus* can resist heat, dryness, and disinfectants since it is able to produce heat-resistant spores [6]. These spores may subsequently germinate into vegetative cells and form biofilms that produce several substances including toxins and enzymes [7,8]. The biofilms allow *B. cereus* to survive in harsh environments. Moreover, the ability to form biofilms in bacteria allows the bacteria to resist antimicrobial agents and disinfectants, as well as to evade the host immune system [9,10].

The *B. cereus* group, also known as *B. cereus sensu lato* is composed of at least 11 species, including *B. cereus sensu stricto* (*B. cereus* s. s.), *B. anthracis*, *B. thuringiensis*, *B. mycoides*, *B. pseudomycoides*, *B. weihenstephanensis*, *B. cytotoxicus*, *B. toyonensis*, *B. gaemokensis*, *B. manliponensis*, and *B. bingmayongensis* [11]. Since the physiological-biological properties and the 16S rRNA sequences of the *B. cereus* group are similar, species of the *B. cereus* group may not be confirmed through traditional detection methods and phenotyping technologies [12]. Through next-generation sequencing technologies, whole bacterial genomes were evaluated, thereby enabling the establishment of moderate to high conservation of bacterial species via numerous proteins and aid in phylogeny identification [13,14].

Whole genome sequencing (WGS) of bacterial pathogens provides genome sequence information of the bacteria of interest, as well as of unknown bacteria, which can aid in revealing phylogenic and evolutionary trends [13,14]. The study of evolutionary trends and bacterial pathogenesis is beneficial for the development of novel antimicrobial agents or new strategies for pathogen control. Through complete genome sequencing of *Bacillus halotolerans*, tolerance potential to drought and salt stress was revealed [15]. Comparative genome analysis of *Bacillus sporothermodurans* allowed for characterization of the genes involved in heat resistance [16]. Key genes with insect resistance functions in *Bacillus thuringiensis* were characterized by complete genome sequencing, thereby providing genetic information for the development of potential bioinsecticides [17]. Some *Bacillus* sp. possess diarrheal toxin genes and virulence genes, which can be harmful to human health. Complete genome sequencing can be used to identify toxin and virulence genes, as well as genes related to biofilm formation in *Bacillus pacificus* isolate from food [18].

In this study, molecular approaches are used to characterize *Bacillus* spp. isolates from food samples with a heat resistance phenotype to identify evolutionary associations as well as to understand their heat resistance mechanisms. WGS analyses were performed in *Bacillus* sp. B48 and *Bacillus* sp. B140, isolates from an omelet with acacia leaves and fried rice, respectively [19]. Their closest phylogenetic neighbor and the genes involved in heat resistance were characterized to better understand this phenotype in *B. cereus* group. Moreover, molecular comparisons of these two isolates with mesophilic strain *B. cereus* ATCC 14579 and thermophilic strain *B. cytotoxicus* NVH 391-98 were also performed. This work provides evidence for the potential genetic basis for adaptation to heat conditions in the *Bacillus* species.

## Methods

### Bacterial strain

*Bacillus* sp. B48 and *Bacillus* sp. B140 isolates were isolated from an omelet with acacia leaves and fried rice [19], respectively. Glycerol stocks of the bacteria, stored at –80ºC, were streaked on nutrient agar plates and incubated overnight at 35 ± 2ºC.

### Thermotolerance of *B. cereus* strains

The thermotolerance of *B. cereus* isolates was determined at a lethal temperature (60°C). The protocol was performed according to Periago et al. [20] with some modifications. Cultures in the mid-exponential growth phase (optical density at 600 nm of 0.5), were exposed to heat treatment at 60°C for 0, 1, 3, 6, 24, and 48 h. Viable counts were monitored by plate counting. Three independent experiments were performed for each isolate.

### Antimicrobial susceptibility tests

The antimicrobial susceptibility of *Bacillus* sp. B48 and *Bacillus* sp. B140 was determined using the Kirby–Bauer disk diffusion method according to standard criteria of the Clinical and Laboratory Standards Institute (CLSI) 2010 [21]. The antimicrobial discs tested in this study were procured from Himedia, Mumbai, India. The antimicrobial agents included ampicillin (AMP, 10 µg), amoxicillin-clavulanic acid (AMC, 20 µg/10 µg), penicillin G (PEN, 10 U), gentamicin (GEN, 10 µg), imipenem (IPM, 10 µg), vancomycin (VAN, 30 µg), chloramphenicol (CHL, 30 µg), ciprofloxacin (CIP, 5 µg), tetracycline (TET, 30 µg), and erythromycin (ERY, 15 µg). The isolates were classified as sensitive (S), intermediate (I), or resistant (R) to each antimicrobial agent based on the zones of inhibition, according to the interpretative criteria for *Staphylococcus* spp., following CLSI guidelines [22]. *Staphylococcus aureus* ATCC 25923 was assigned as a control strain for these antimicrobial susceptibility tests.

### WGS, assembly, and annotation

Genomic DNA (gDNA) was extracted and purified using a GF-1 Bacterial DNA Extraction Kit (Vivantis Technologies, Selangor, Malaysia) according to the manufacturer’s recommended protocol. The quality of the gDNA was determined by agarose gel electrophoresis and spectrophotometry. The gDNA libraries were prepared using a QIAGEN FX kit (Qiagen, Valencia, CA, USA). The quality and quantity of the indexed libraries were verified using an QIAxcel advance and a Denovix fluorometer. Then, the libraries were pooled in equimolar quantities and sequenced on an Illumina Miseq 2X250 bp paired-end (Illumina Inc., San Diego, CA, USA). Raw read quality was determined using the FASTQC software. Adaptors and poor-quality reads were removed using Trimmomatic, and the filtered reads were used as inputs for the Unicycler genome assembly program with default parameters [23].

The genomes of *Bacillus* sp. B48 and B140 were annotated using the Rapid Annotation using Subsystem Technology tool kit (RASTtk) in PATRIC (Pathosystems Resource Integration Center) [24]. Graphical circular genome maps were constructed by using the circular viewer in PATRIC. The virulence factors and antimicrobial resistance were also identified using PATRIC. The predicted coding sequence (CDS) was annotated from clusters of orthologous groups of proteins (COG) and Kyoto Encyclopedia of Genes and Genomes (KEGG) databases using the alignment tool, BlastKOALA (https://www.kegg.jp/blastkoala/) and eggNOG-mapper. A Venn diagram was used to compare the CDS of *Bacillus* sp. B48 and *Bacillus* sp. B140 with those of *B. cereus* ATCC14579 and *B. cytotoxicus* NVH 391-98. The whole genome sequences of *Bacillus* sp. B48 and *Bacillus* sp. B140 were compared with that of *B. cereus* ATCC14579 and *B. cytotoxicus* NVH 391-98 using Mauve 2.3.1. A WGS-based phylogenetic tree was constructed using the CodonTree method within PATRIC webserver, which used the BV-BRC global Protein Families (PGFams) as homology groups [25]. The aligned protein and coding DNA were used to build the main tree with the RAxML analysis. Support values for the phylogenetic tree were generated using 100 rounds of the rapid bootstrapping option of RAxML [26]. FigTree (http://tree.bio.ed.ac.uk/software/figtree/) and iTOL were used for tree visualization [27]. Sequences of prophage Clp protease-like proteins from *Bacillus* sp. B48 and B140 were aligned using the Clustal Omega tool (https://www.ebi.ac.uk/Tools/msa/clustalo/).

### Nucleotide sequence accession numbers

The draft genomes of *Bacillus* sp. B48 and B140 were deposited in the NCBI SRA under the accession numbers SRR23924657 and SRR23924656, respectively. The BioSample accession for *Bacillus* sp. B48 and B140 were SAMN33833737 and SAMN33833738, respectively. The BioProject ID in GenBank is PRJNA946912.

## Results

### Thermotolerance of *Bacillus* sp. B48 and *Bacillus* sp. B.140

**Fig. 1.**
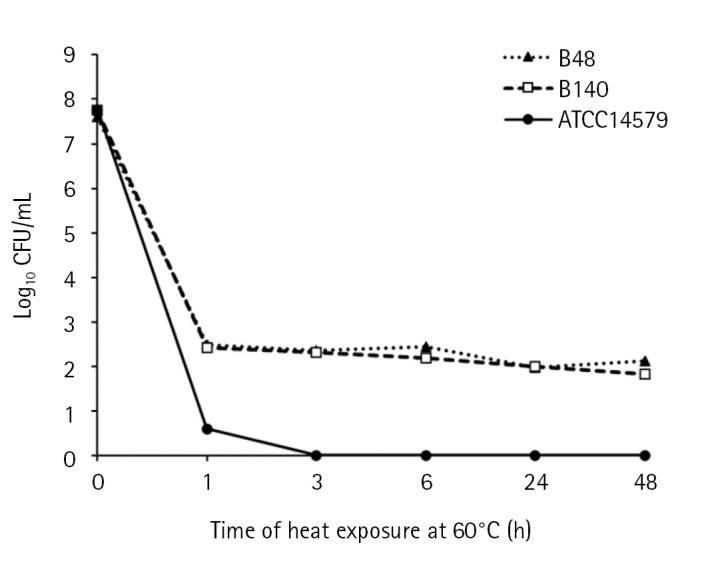
Thermotolerance of *Bacillus* sp. B48 and *Bacillus* sp. B140. Log-phase bacterial cells were exposed to heat at 60 ºC for 0, 1, 3, 6, 24, and 48 h. Colony counts were performed after incubation on nutrient agar plates at 35 ± 2ºC overnight. Three independent experiments were performed for each isolate. B48, *Bacillus* sp. B48; B140, *Bacillus* sp. B140; ATCC14579, *B. cereus* ATCC14579.

The thermotolerance of *B. cereus* isolates was observed at a lethal temperature (60ºC) compared to mesophilic strain *B. cereus* ATCC 14579 as the control (Fig. 1). The colony counts of *B. cereus* ATCC14579 were dramatically decreased upon exposure to heat at 60ºC for 1 h and colonies were undetectable after 3 h. The colony counts of *Bacillus* sp. B48 and *Bacillus* sp. B140 decreased from log_10_ = 8 CFU/mL to approximately 2 after heat treatment for 1 h, and the constant colony count was observed even up to 48 h.

### Antimicrobial resistance profiles of *Bacillus* sp. B48 and *Bacillus* sp. B140

*Bacillus* sp. B48 and *Bacillus* sp. B140 were assessed for antimicrobial resistance to 10 antimicrobial agents (Table 1). Both isolates were susceptible to most of the tested antimicrobial agents, including GEN (26.6 ± 0.7 mm for *Bacillus* sp. B48 and 26.4 ± 0.4 mm for *Bacillus* sp. B140), IPM (37.3 ± 0.7 mm for *Bacillus* sp. B48 and 23.8 ± 1.9 mm for *Bacillus* sp. B140), VAN (23.1 ± 1.2 mm for *Bacillus* sp. B48 and 22.7 ± 0.6 mm for *Bacillus* sp. B140), CHL (31.5 ± 0.9 mm for *Bacillus* sp. B48 and 25.2 ± 0.7 mm for *Bacillus* sp. B140), CIP (32.0 ± 1.3 mm for *Bacillus* sp. B48 and 31.7 ± 0.6 mm for *Bacillus* sp. B140), TET (27.6 ± 1.1 mm for *Bacillus* sp. B48 and 27.5 ± 0.5 mm for *Bacillus* sp. B140), and ERY (29.2 ± 0.7 mm for *Bacillus* sp. B48 and 29.1 ± 0.9 mm for *Bacillus* sp. B140). However, both isolates were resistant to β-lactam antibiotics including AMP (10.3 ± 0.4 mm for *Bacillus* sp. B48 and 9.5 ± 0.6 mm for *Bacillus* sp. B140), AMC (5.9 ± 0.1 mm for *Bacillus* sp. B48 and 6.1 ± 0.2 mm for *Bacillus* sp. B140), and PEN (7.1 ± 0.5 mm for *Bacillus* sp. B48 and 7.8 ± 1.7 mm for *Bacillus* sp. B140).

### Genetic features of *Bacillus* sp. B48 and *Bacillus* sp. B140

**Fig. 2.**
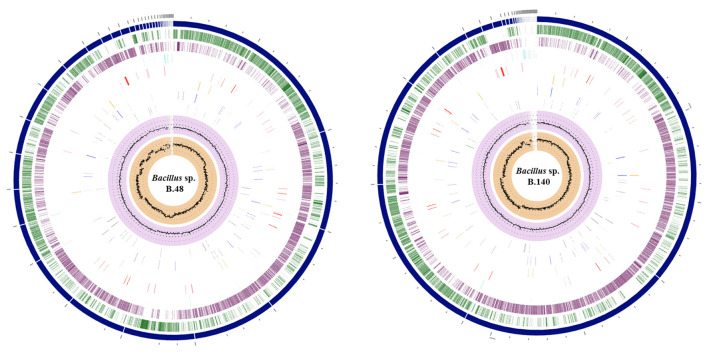
Genomic circular diagram of *Bacillus* sp. B48 and *Bacillus* sp. B140. A genome circular diagram was reconstructed using the circular viewer of PATRIC, from the outer circle to the inner circle: contigs, forward coding sequence (CDS), reverse CDS, non-CDS features, AMR genes, VF genes, transporters, and drug targets. The two inner tracks are GC content and GC skew.

The genomic features and annotation information are summarized in Table 2. The sequences of the genome drafts of *Bacillus* sp. B48 and *Bacillus* sp. B140 had estimated length of 5,533,408 bp and 5,279,040 bp with GC contents of 34.91% and 35.01%, respectively. The coding sequences were 5,705 bp for *Bacillus* sp. B48 and 5,468 bp for *Bacillus* sp. B140. The circular representation of the *Bacillus* sp. B48 and *Bacillus* sp. B140 draft genomes are shown in Fig. 2. The specialty genes of *Bacillus* sp. B48 and B140 including those involved in virulence factors and antibiotic resistance are summarized in Table 3.

**Fig. 3.**
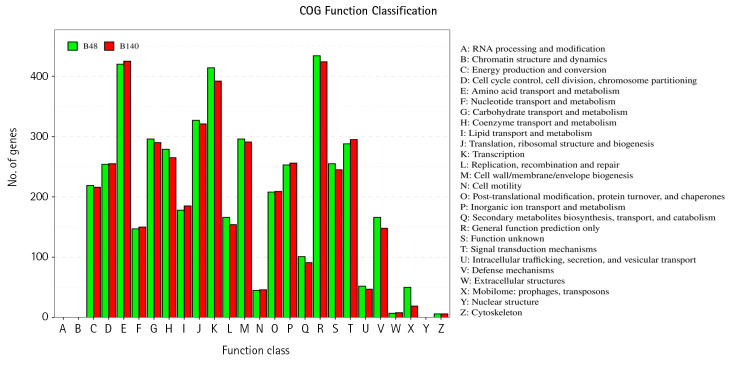
The clusters of orthologous groups of proteins (COG) function annotation of *Bacillus* sp. B48 (B48) and *Bacillus* sp. B140 (B140). The X-axis represents the functional classification of COG. The Y-axis represents the number of genes annotated in each classification. The details of each classification are illustrated on the right.

**Fig. 4.**
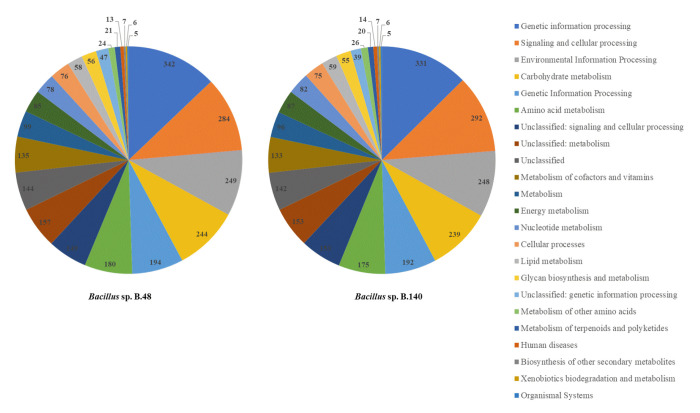
Genome annotation of *Bacillus* sp. B48 and *Bacillus* sp. B140 by BlastKOALA Kyoto Encyclopedia of Genes and Genomes (KEGG). The total number of proteins assigned to the most abundant categories are illustrated.

The genomes of *Bacillus* sp. B48 and *Bacillus* sp. *Bacillus* sp. B140 were characterized using eggNOG database to analyze the COGs of proteins with functional annotations (Fig. 3). The majority of the annotated genes were classified as “Amino acid transport and metabolism” (E category), “General function prediction only” (R category), and “Transcription” (K category). These categories were similarly distributed in both strains. Functional annotation of both bacterial genomes was performed by the BlastKOALA tool on the KEGG Orthology (KO) database. A number of genes were classified as genetic information processing, signaling and cellular processing, environmental information processing, carbohydrate metabolism, and amino acid metabolism which being the most represented categories (Fig. 4).

### Phylogenetic analysis

**Fig. 5.**
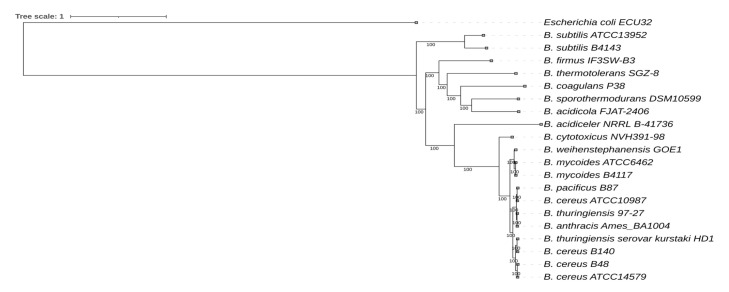
Whole genome sequencing–based phylogenetic tree of *Bacillus* sp. B48 and *Bacillus* sp. B140. The codon tree method selects single-copy PATRIC PGFams. Aligned proteins and coding DNA from single-copy genes were analyzed using the RAxML program. FigTree and iTOL were used for tree visualization. *Escherichia coli* was used as the outgroup.

The phylogenetic relationship of *Bacillus* species is illustrated in Fig. 5. *Bacillus* sp. B48 was found to be closely related to *B. cereus* while *Bacillus* sp. B140 was found to be closely related to *B. thuringiensis*.

### Analysis of orthologous gene clusters

**Fig. 6.**
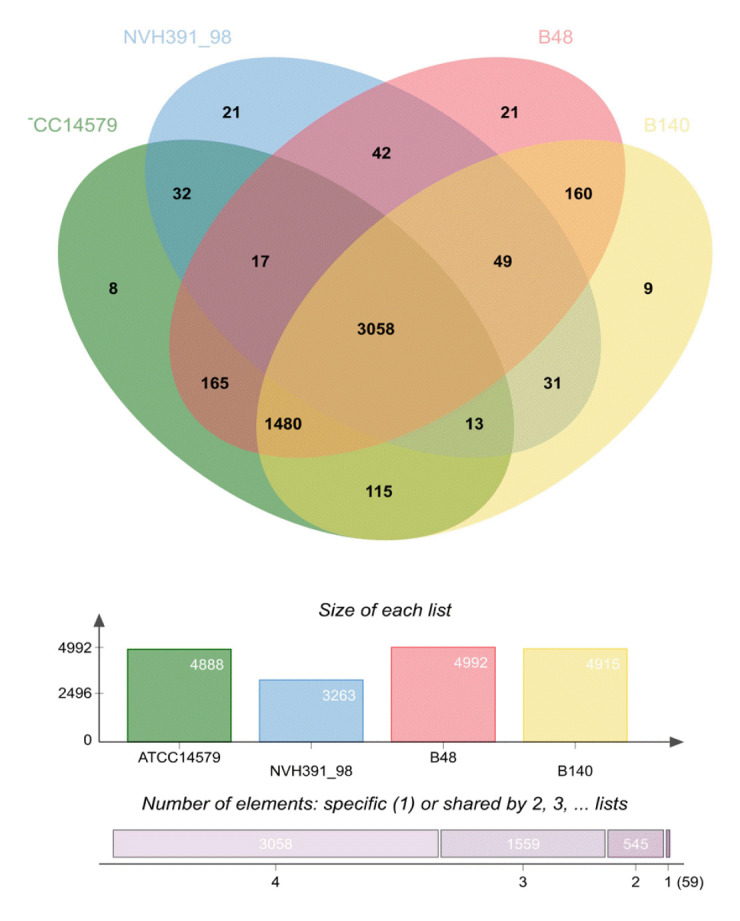
Venn diagram of *Bacillus* sp. B48, *Bacillus* sp. B140, *B. cereus* ATCC14579, and *B. cytotoxicus* NVH 391-98. Whole genome comparison and annotation of orthologous gene clusters were performed using OrthoVenn2.

Analysis of orthologous gene clusters using OrthoVenn2 demonstrated that a core of 3,058 orthologous genes was shared among the genomes of *Bacillus* sp. B48, *Bacillus* sp. B140, *B. cereus* ATCC14579, and *B. cytotoxicus* NVH 391-98 (Fig. 6). There were 506, 339, 382, and 541 singleton gene clusters present in *Bacillus* sp. B48, *Bacillus* sp. B140, *B. cereus* ATCC14579, and *B. cytotoxicus* NVH 391-98, respectively (Table 4). The genomes of *Bacillus* sp. B48, *Bacillus* sp. B140, *B. cereus* ATCC14579, and *B. cytotoxicus* NVH 391-98 contained 21, 9, 8, and 21 unique genes, respectively (Supplementary Table 1).

### Analysis of protein clusters involved in heat resistance in *Bacillus* species

**Fig. 7.**
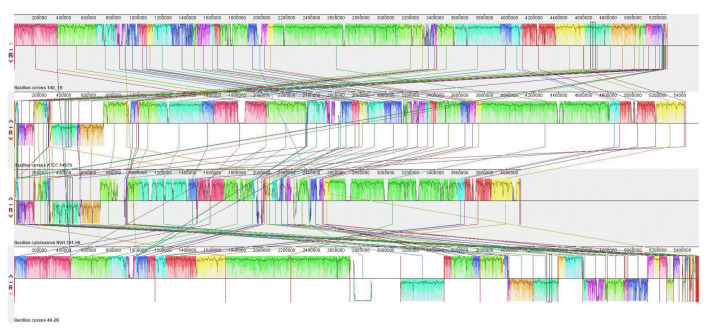
Whole genome alignment of *Bacillus* sp. B48, *Bacillus* sp. B140, *B. cereus* ATCC14579, and *B. cytotoxicus* NVH 391-98 using the Mauve program. Strain names are indicated on the left. Regions with homologous sequences are illustrated with the same-colored squares.

**Fig. 8.**
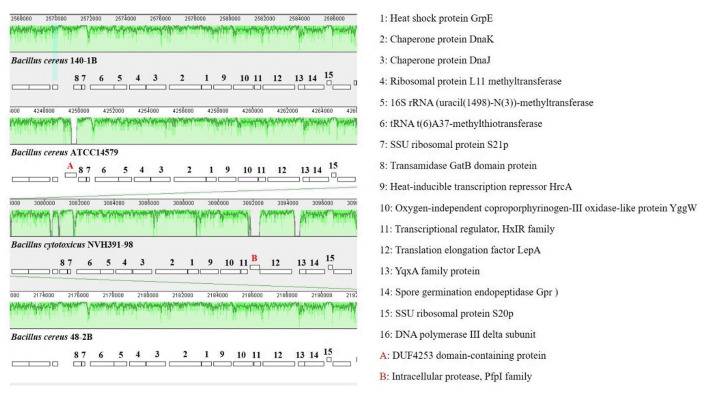
Comparison of chromosomal regions of the heat shock protein GrpE of *Bacillus* sp. B48, *Bacillus* sp. B140, *B. cereus* ATCC14579, and *B. cytotoxicus* NVH 391-98 in the Mauve program. The details of each number are described on the right.

Whole genome alignments of *Bacillus* sp. B48, *Bacillus* sp. B140, *B. cereus* ATCC14579, and *B. cytotoxicus* NVH 391-98 revealed the existence of numerous homologous regions (Fig. 7). The genomic arrangement of the heat shock protein *grpE*, as well as associated genes responsible for heat shock in the *dnaK* gene cluster, are shown in Fig. 8. The similarity of chromosomal regions of the heat shock proteins was observed in all four strains except for the presence of DUF4253 domain-containing protein in the chromosome of *B. cereus* ATCC14579 and the intracellular protease, PfpI family, in the chromosome of *B. cytotoxicus* NVH 391-98.

**Fig. 9.**
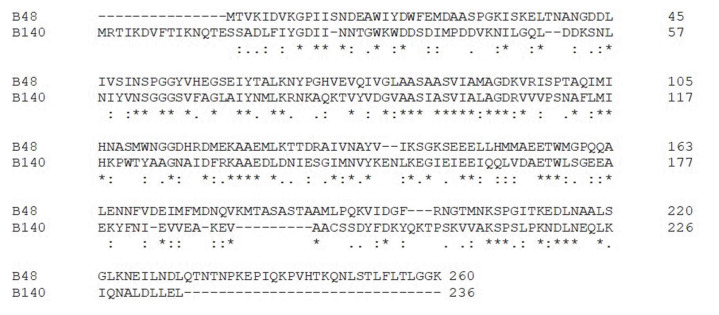
Amino acid sequence alignment of the prophage Clp protease-like proteins of *Bacillus* sp. B48 (B48) and *Bacillus* sp. B140 (B140). Sequences alignments were performed using Clustal Omega.

PATRIC analysis showed that the prophage Clp protease-like proteins were present in the genome of *Bacillus* sp. B48 and *Bacillus* sp. B140. Fig. 9 shows the amino acid sequence alignments of the prophage Clp protease-like proteins of *Bacillus* sp. B48 and *Bacillus* sp. B140 with low similarity.

## Discussion

In this study, we genetically characterized food-isolated strains of *Bacillus* sp. B48 and *Bacillus* sp. B140. Both strains are resistant to heat treatment at 60°C for at least 48 h. Therefore, it is interesting to study their genomic profiles, especially those related to heat responses such as heat shock gene clusters. Several emerging highly heat-resistant *Bacillus* species have been previously classified including *B. thermoamylovorans*, *B. sporothermodurans*, and *B. cytotoxicus* NVH 391-98 [16,28,29]. *B. sporothermodurans* can significantly affect the quality of heat-processed foods since it is able to form heat-resistant spores that can survive in foods treated with ultra-high temperatures [16]. *B. thermoamylovorans* is an emerging highly heat-resistant *Bacillus* species isolated from milk [28]. *B. cytotoxicus* NVH 391-98, a thermotolerant species of the *B. cereus* group, was isolated during a severe food poisoning outbreak in France in 1998 [29]. However, phylogenetic analysis suggested that both *Bacillus* sp. B48 and *Bacillus* sp. B140 are not closely related to *B. thermoamylovorans*, *B. sporothermodurans*, or *B. cytotoxicus* NVH 391-98. Based on whole genome phylogenetic analysis, *Bacillus* sp. B48 and *Bacillus* sp. B140 were found to be closely related to *B. cereus* and *B. thuringiensis*, respectively. Therefore, the categorization of bacterial inhabitants is based on sequence similarity threshold rather than biological relevance [16,30].

Heat shock proteins (HSPs) are responsible for heat shock treatment including thermal food processing. Several conditions other than heat shock can induce HSP expression, these include cold, ultraviolet light, and during cell healing [31]. Major HSPs that have been reported in prokaryotes include GroES, GrpE, DnaJ, GroEL, DnaK, HtpG, ClpB, ClpA, and ClpX [31-35]. Most of these HSPs are present in *B. cereus* ATCC14579 and *B. cytotoxicus* NVH 391-98, as well as in *Bacillus* sp. B48 and *Bacillus* sp. B140. These molecular chaperones are important because they prevent protein aggregation during heat stress [16,20]. The crucial process of high heat resistance in *Bacillus* spp. during periods of stress and sporulation requires ClpA, ClpC, ClpE, ClpP, ClpQ, ClpX, and ClpY [36]. In various prokaryotes, the ClpB, ClpA, and ClpX protein complex modulates the tolerance to extreme temperatures by remodeling and degrading aggregated proteins [37]. ClpB, which was identified in *B. subtilis*, *B. sporothermodurans*, and *B. oleronius*, works together with the chaperones DnaJ, DnaK, and GrpE to repair heat-induced protein damage [38]. In *B. subtilis*, ClpX together with ClpP play a role in cell survival at thermal stress under limiting levels of the DnaK system [39]. We found that both *Bacillus* sp. B48 and *Bacillus* sp. B140, but not *B. cereus* ATCC14579, contain prophage Clp protease-like proteins. These proteins may be involved in the heat-tolerant phenotype in both isolates. However, it is necessary to further characterize the role of these proteins especially relating to the heat-resistant phenotype.

HrcA is a heat-inducible transcription repressor detected in *B. subtilis* and *B. sporothermodurans*. DnaK, DnaJ, and GrpE are involved in the repair of heat-induced protein damage by forming a cellular chaperone machinery [33]. GrpE is the chaperone moiety of the *dnaK* operon that works together with GroEL for the renaturation of heat-denatured proteins [33]. Many of these proteins are shared amongst the *Bacillus* species and they are also homologs of each other, with some variations in the amino acid sequences. All four strains mentioned in this study also possess these proteins with some variations in amino acid sequences. However, the DUF4253 domain-containing protein is present only in *B. cereus* ATCC14579 but not in *Bacillus* sp. B48, *Bacillus* sp. B140 or *B. cytotoxicus* NVH 391-98. Four domains of unknown function including DUF438, DUF1524, DUF1957, and DUF3458_C, have been hypothesized to play roles in temperature adaptation in both archaea and bacteria [40]. Therefore, the function of this protein in heat response in *Bacillus* species needs to be further characterized.

In conclusion, *B. cereus*, a foodborne pathogen that can cause diarrheal or emetic food poisoning, can grow and survive under varied temperatures with diverse heat-responsive mechanisms, especially those that require HSPs. In this study, a genomic analysis of food-isolated *Bacillus* sp. B48 and *Bacillus* sp. B140 with thermotolerant phenotype revealed the presence of prophage Clp protease-like proteins with low amino acid sequences similarities. This protein was not present in the genome of the mesophilic strain, *B. cereus* ATCC14579. Further experimental analysis in terms of gene knockout and protein characterization is required to confirm the function of prophage Clp protease-like proteins relating to heat tolerant phenotype of the bacteria, especially in *Bacillus* species.

## Figures and Tables

**Table 1.** Antimicrobial susceptibility testing of *Bacillus* sp. B48 and *Bacillus* sp. B140

Category	Antimicrobial agent	Isolates	
		*Bacillus* sp. B48	*Bacillus* sp. B140
β-lactam	Ampicillin (AMP, 10 µg)	R	R
	Amoxicillin-clavulanic acid (AMC, 20 µg/10 µg)	R	R
	Penicillin (PEN, 10 U)	R	R
Aminoglycosides	Gentamicin (GEN, 10 µg)	S	S
Carbapenems	Imipenem (IPM, 10 µg)	S	S
Glycopeptides	Vancomycin (VAN, 30 µg)	S	S
Phenicols	Chloramphenicol (CHL, 30 µg)	S	S
Fluoroquinolones	Ciprofloxacin (CIP, 5 µg)	S	S
Tetracyclines	Tetracycline (TET, 30 µg)	S	S
Macrolides	Erythromycin (ERY, 15 µg)	S	S

S, sensitive, R, resistant.

**Table 2.** Genomic features and annotation information of the chromosome of *Bacillus* sp. B48 and *Bacillus* sp. B140

Genome feature	*Bacillus* sp. B48	*Bacillus* sp. B140
Genome length (bp)	5,533,408	5,279,040
Protein-coding genes	5,705	5,468
GC content (%)	34.91	35.01
No. of tRNAs	54	71
No. of rRNAs	4	6
Contigs	38	35

GC content, guanine and cytosine content; tRNA, transfer RNA; rRNA, ribosomal RNA

**Table 3.** Specialty genes in genomic sequence of *Bacillus* sp. B48 and *Bacillus* sp. B140 annotated using Subsystem Technology tool kit (RASTtk) in PATRIC

	*Bacillus* sp. B48	*Bacillus* sp. B140
Virulence factor		
Victors database	11	12
	(*phnX*: phosphonoacetaldehyde hydrolase (EC 3.11.1.1); *GBAA4766*: ferrichrome-binding periplasmic protein precursor (TC 3.A.1.14.3)); *alo*: thiol-activated cytolysin; *sodA2*: superoxide dismutase [Mn] (EC 1.15.1.1; *sigB*: RNA polymerase sigma factor SigB; *sodC*: superoxide dismutase [Cu-Zn] precursor (EC 1.15.1.1); *sodA1*: superoxide dismutase [Mn] (EC 1.15.1.1); *clpX*: ATP-dependent Clp protease ATP-binding subunit ClpX; *asbA*: anthrachelin biosynthesis protein AsbA @ Siderophore synthetase superfamily, group A @ Siderophore synthetase large component, acetyltransferase; *nos*: nitric oxide synthase oxygenase (EC 1.-.-.-); *codY*: GTP-sensing transcriptional pleiotropic repressor CodY)	(*phnX*: phosphonoacetaldehyde hydrolase (EC 3.11.1.1); *codY*: GTP-sensing transcriptional pleiotropic repressor CodY; *sodA1*- Superoxide dismutase [Mn] (EC 1.15.1.1);* GBAA4766*: ferrichrome-binding periplasmic protein precursor (TC 3.A.1.14.3); *sigB*: RNA polymerase sigma factor SigB; *alo*: thiol-activated cytolysin; *asbA*: anthrachelin biosynthesis protein AsbA @ Siderophore synthetase superfamily, group A @ Siderophore synthetase large component, acetyltransferase; *clpX*: ATP-dependent Clp protease ATP-binding subunit ClpX; *sodC*: superoxide dismutase [Cu-Zn] precursor (EC 1.15.1.1); *recA*: RecA protein; *nos*: nitric oxide synthase oxygenase (EC 1.-.-.-); *sodA2*: superoxide dismutase [Mn] (EC 1.15.1.1))
VFDB database	9	9
	(*BAS3109*: thiol-activated cytolysin; *cytK*: cytolytic pore-forming protein => cytotoxin K; *hblC*: hemolysin BL lytic component L2; *hblA*: hypothetical protein; *inhA*: immune inhibitor A, metalloprotease (EC 3.4.24.-); *hblD*: hypothetical protein; *nheC*: enterotoxin C; *nheA*: non-hemolytic enterotoxin A; *nheB*: non-hemolytic enterotoxin lytic component L1)	(*hblA*: hypothetical protein; *cytK*: cytolytic pore-forming protein => cytotoxin K; *nheA*: non-hemolytic enterotoxin A; *nheB*: non-hemolytic enterotoxin lytic component L1; *nheC*: enterotoxin C; *hblD*: hypothetical protein; *hblC*: hypothetical protein; *BAS3109*: thiol-activated cytolysin; *inhA*: immune inhibitor A, metalloprotease (EC 3.4.24.-))
Antibiotic resistance		
CARD database	6	6
	(*bcrA*: bacitracin efflux ABC transporter, ATP-binding protein BcrA; *BcI*: class A beta-lactamase (EC 3.5.2.6); *FosB*: fosfomycin resistance protein FosB; *lsaB*: ABC-F type ribosomal protection protein => Lsa(B); translation elongation factor Tu; *BcII*: subclass B1 beta-lactamase (EC 3.5.2.6) => BcII family)	(*dfrE*: thymidylate synthase (EC 2.1.1.45); *FosB*: fosfomycin resistance protein FosB; *BcI*: class A beta-lactamase (EC 3.5.2.6); *BcII*: subclass B1 beta-lactamase (EC 3.5.2.6) => BcII family; translation elongation factor Tu; *lsaB*: ABC-F type ribosomal protection protein => Lsa(B))
NDARO database	4	4
	(Subclass B1 beta-lactamase (EC 3.5.2.6) => BcII family; fosfomycin resistance protein FosB; Class A beta-lactamase (EC 3.5.2.6); ABC-F type ribosomal protection protein => Lsa(B))	(class A beta-lactamase (EC 3.5.2.6); fosfomycin resistance protein FosB; ABC-F type ribosomal protection protein => Lsa(B); subclass B1 beta-lactamase (EC 3.5.2.6) => BcII family)

**Table 4.** Comparative analysis of gene clusters using OrthoVenn2

Species	Proteins	Clusters	Singletons
*Bacillus* sp. B48	5,550	4,992	506
*Bacillus* sp. B140	5,294	4,915	339
*B. cereus* ATCC14579	5,316	4,888	382
*B. cytotoxicus* NVH 391-98	3,855	3,263	541

## Supplementary Materials

Supplementary Table 1. Cluster list of the unique genes observed in *Bacillus* sp. B48 and *Bacillus* sp. B104 genomes using OrthoVenn2
