# Supplementary material for: Whole genome sequence analyses of thermotolerant Bacillus sp. isolates from food
Source: Genomics Inform. 2023 Sep 27;21(3):e35. doi: 10.5808/gi.23030 (PMC10584648; doi:10.5808/gi.23030)
Supplement: Supplementary Table 1. — Cluster list of the unique genes observed in Bacillus sp. B48 and Bacillus sp. B104 genomes using OrthoVenn2 [file gi-23030-Supplementary-Table-1.pdf]

Cluster list

B48	# cluster_name	protein_number	swiss_prot_id	go_annotation
1	cluster3085	3	N/A	N/A
2	cluster4643	2	N/A	N/A
3	cluster4644	2	N/A	N/A
4	cluster4645	2	O31178	GO:0003700; F:DNA-binding transcription factor activity; IEA:InterPro
5	cluster4646	2	N/A	N/A
6	cluster4647	2	N/A	N/A
7	cluster4648	2	N/A	N/A
8	cluster4649	2	N/A	N/A
9	cluster4650	2	N/A	N/A
10	cluster4651	2	N/A	N/A
11	cluster4652	2	N/A	N/A
12	cluster4653	2	N/A	N/A
13	cluster4654	2	P13772	GO:0043565; F:sequence-specific DNA binding; IEA:InterPro
14	cluster4655	2	N/A	N/A
15	cluster4656	2	L7N653	GO:0009253; P:peptidoglycan catabolic process; IEA:InterPro
16	cluster4657	2	N/A	N/A
17	cluster4658	2	N/A	N/A
18	cluster4659	2	N/A	N/A
19	cluster4660	2	N/A	N/A
20	cluster4661	2	N/A	N/A
21	cluster4662	2	N/A	N/A

B140	# cluster_name	protein_number	swiss_prot_id	go_annotation
1	cluster19	6	N/A	N/A
2	cluster5161	2	P54232	N/A
3	cluster5162	2	N/A	N/A
4	cluster5163	2	N/A	N/A
5	cluster5164	2	N/A	N/A
6	cluster5165	2	N/A	N/A
7	cluster5166	2	N/A	N/A
8	cluster5167	2	N/A	N/A
9	cluster5168	2	N/A	N/A
